# Trauma-informed Care Training in Trauma and Emergency Medicine: A Review of the Existing Curricula

**DOI:** 10.5811/westjem.18394

**Published:** 2024-04-08

**Authors:** Cecelia Morra, Kevin Nguyen, Rita Sieracki, Ashley Pavlic, Courtney Barry

**Affiliations:** * Medical College of Wisconsin, Milwaukee, Wisconsin; † Medical College of Wisconsin, Department of Emergency Medicine, Milwaukee, Wisconsin; ‡ Medical College of Wisconsin, Department of Psychiatry and Behavioral Medicine, Milwaukee, Wisconsin

## Abstract

**Background and Objectives:**

Greater lifetime exposure to psychological trauma correlates with a higher number of health comorbidities and negative health outcomes. However, physicians often are not specifically trained in how to care for patients with trauma, especially in acute care settings. Our objective was to identify implemented trauma-informed care (TIC) training protocols for emergency and/or trauma service physicians that have both sufficient detail that they can be adapted and outcome data indicating positive impact.

**Methods:**

We conducted a comprehensive literature search in MEDLINE (Ovid), Scopus, PsycInfo, Web of Science, Cochrane Library, Ebsco’s Academic Search Premier, and MedEdPORTAL. Inclusion criteria were EM and trauma service clinicians (medical doctors, physician assistants and nurse practitioners, residents), adult and/or pediatric patients, and training evaluation. Evaluation was based on the Kirkpatrick Model.

**Results:**

We screened 2,280 unique articles and identified two different training protocols. Results demonstrated the training included patient-centered communication and interprofessional collaboration. One curriculum demonstrated that targeted outcomes were due to the training (Level 4). Both curricula received overall positive reactions (Level 1) and illustrated behavioral change (Level 3). Neither were found to specifically illustrate learning due to the training (Level 2).

**Conclusion:**

Study findings from our review show a paucity of published TIC training protocols that demonstrate positive impact and are described sufficiently to be adopted broadly. Current training protocols demonstrated an increasing comfort level with the TIC approach, integration into current practices, and referrals to trauma intervention specialists.

Population Health Research Capsule What do we already know about this issue?

*Many patients who access emergency care have a history of psychological trauma. Best practices recommend a trauma-informed care (TIC) approach.*

What was the research question?

*What TIC training protocols have shown a positive impact for emergency and trauma clinicians?*

What was the major quantitative finding of the study? Major comparison with p-value and confidence interval.

*Only two TIC curricula in the literature show positive impact and are reproducible.*

How does this improve population health?

*The limited existing curricula show that targeted TIC training increases clinician use of TIC practices and improves patient outcomes and satisfaction.*

## INTRODUCTION

Greater psychological trauma exposure within one’s lifetime correlates with an increased number of health comorbidities and negative health outcomes.^1^ Childhood exposures to trauma are linked to increased health risks in adulthood for substance use disorder, depression, obesity, heart disease, cancer, and more. Experiencing trauma is often thought of as a rare occurrence, but the foundational adverse childhood experiences (ACE) study has shown how common and pervasive traumatic events are within the US. The study investigated different categories of childhood trauma that included physical/sexual/emotional abuse, parental incarceration, and parental drug use. More than half of the participants reported at least one ACE, and 25% reported more than two categories of ACEs. From 2011–2015, the state of Wisconsin ran the Behavioral Risk Factor Survey, which found that 57% of the 25,518 adult participants reported one or more ACEs.^2^

Many studies recommend screening for ACEs in the emergency department (ED), but this has not become common practice.^3^ The ACE questionnaire remains the most common tool used for such screening^1^; however, more recent research has suggested that trauma-informed care (TIC) should be applied in all patient interactions because patients with a history of trauma infrequently classify themselves as such.^4^ Practicing with the assumption that each patient has experienced some form of trauma allows the healthcare team to avoid re-traumatization, or the re-experiencing of a prior trauma when exposed to a new traumatic event, and to deliver compassionate and patient-centered care, which is a critical piece in TIC.^5^^–^^7^

Because a patient’s first contact with the healthcare system is often in the acute care setting, it is crucial that these clinicians are equipped with the appropriate resources and knowledge to provide TIC.^5^^,^^8^^,^^9^ This encourages them to use a more mindful approach to assessing patients. Studies have indicated that 11–61% of ED patients present with a trauma history and 20% of patients at admission report suffering from acute emotional distress.^10^^,^^11^ In these acute care settings, there are multiple scenarios in which re-traumatization can occur. For example, although it is not surprising to find that restraint use on a patient can be harmful, studies suggest that even a routine physical exam without verbal cues can unintentionally re-traumatize a patient.^12^^,^^13^ Such events can cause patients to withdraw from the healthcare interaction and decision-making, which leads to a portrayal of patient non-adherence. Furthermore, patients with trauma history are less likely to seek out a primary care physician, instead relying on the ED for treatment.^14^^,^^15^ Thus, if TIC is not practiced in these settings, long-term health outcomes are impaired and morbidity is increased. It is essential that medical staff be trained in trauma-informed practices to provide high-quality care and promote healing.

**Figure 1.**
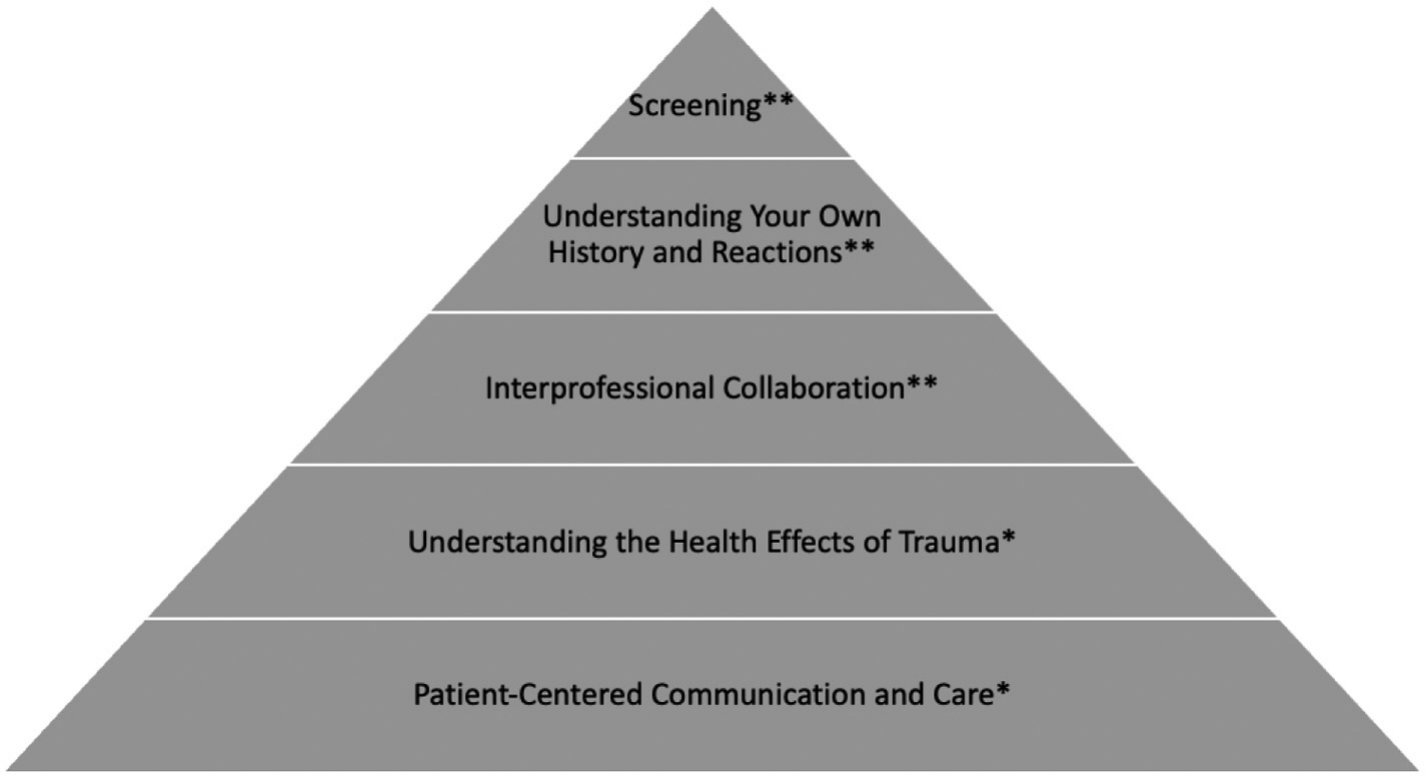
Trauma-informed care pyramid adapted from Raja et al (2015). Universal trauma precautions*; trauma-specific care.**^16^

The TIC pyramid outlines five overarching principles: 1) patient-centered communication and care; 2) understanding the health effects of trauma; 3) interprofessional collaboration; 4) understanding your own history and reactions; and 5) screening, including universal trauma precautions and trauma-specific strategies (Figure 1).^16^ The first two principles are universal precautions that foster trust and rapport and can be used without establishing a patient’s trauma history. The remaining three principles are specific for when the clinician knows the patient has experienced trauma.

The positive impact of TIC in both acute care and primary care settings has been well documented in the literature.^9^^,^^17^^–^^23^ The use of TIC has been associated with improved childhood and family adjustment during periods of increased adversity, enhanced health outcomes, increased satisfaction with care, and better mental health outcomes, with decreased substance abuse rates and reduced post-traumatic stress disorder symptoms.^22^

Practicing TIC can also decrease the psychological and emotional burden on the healthcare team.^10^^,^^23^ Frequent occupational exposure to the trauma experiences of others is considered secondary trauma and is thought to have a cumulative effect on clinician well-being, resulting in greater distress over time.^24^ The impact of these experiences has been described as clinician burnout, compassion fatigue, and secondary traumatic stress. Clinician burnout has been shown to lead to poor sleep, distraction, and defensiveness, among other physical and psychological ramifications.^23^ These reactions impair a clinician’s ability to deliver care, increasing the likelihood of medical errors and making patients feel less safe.^23^ Practicing TIC allows the healthcare team to not only identify and attend to a patient’s prior trauma, mitigate new trauma due to current medical care, and better understand the reactions and behaviors of patients and their families, but also encourages the medical team to recognize their own history with trauma.

Our aim in this scoping review was to identify TIC training protocols designed for US EDs and trauma services that have demonstrated positive impact in order to develop a new training protocol to be used in these settings. Identifying implemented training will assist clinicians, healthcare practices, and teaching programs interested in improving knowledge and clinical practice to address trauma. Reviewing existing training protocols will facilitate adaptation and development of future training to further improve patient care.

## METHODS

### Literature Search

**Figure 2.**
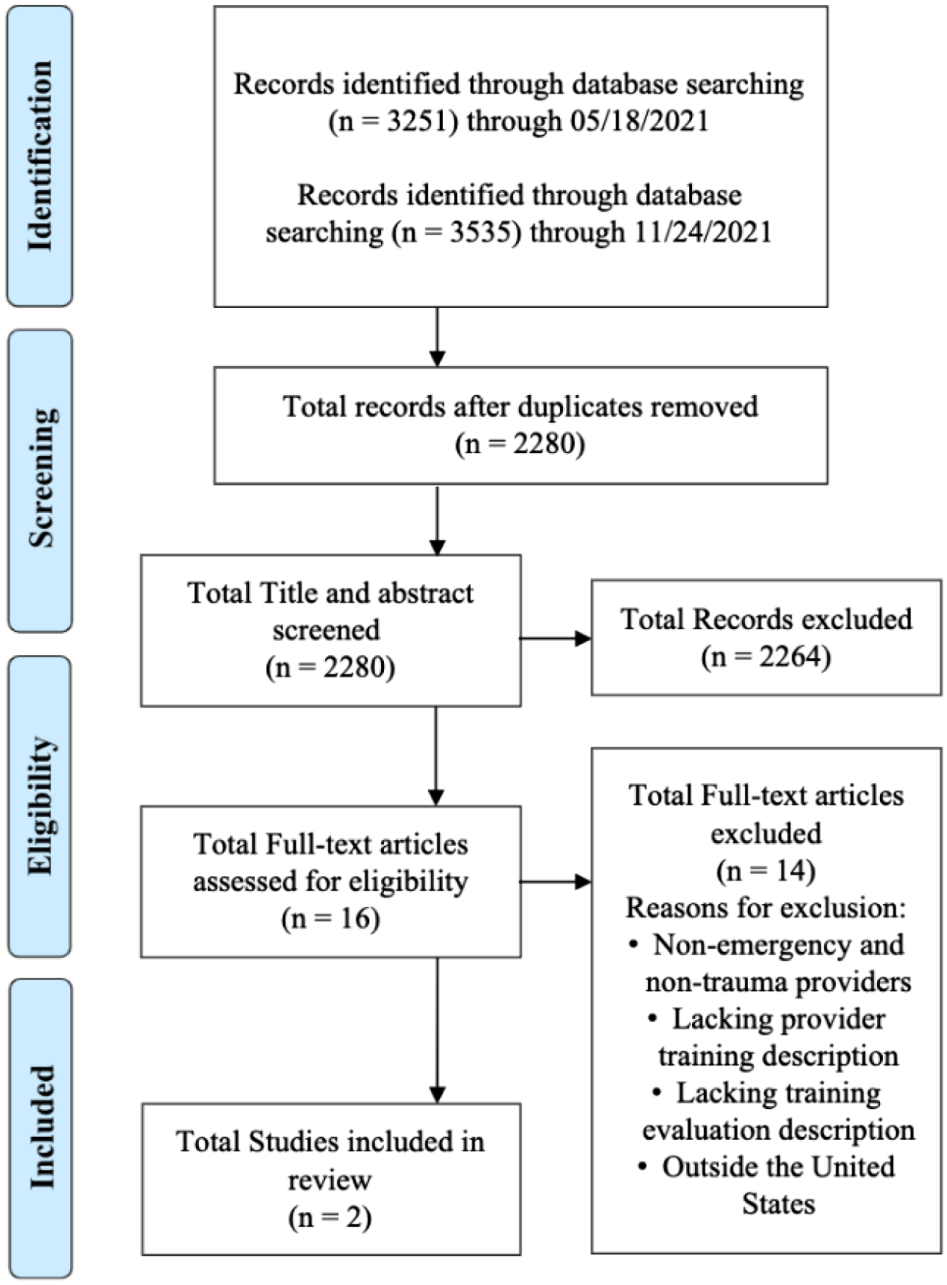
Source selection process.

We used the PRISMA Extension for Scoping Reviews (PRISMA-ScR) checklist as a reporting guide for this review.^25^ Furthermore, we modeled this paper from a previous published paper on a scoping review on TIC within the primary care setting.^21^ A comprehensive literature search was developed by a medical librarian and peer reviewed using the PRESS guideline.^26^ Searches were conducted in MEDLINE (Ovid), Scopus, PsycInfo, Web of Science, Cochrane Library, Ebsco’s Academic Search Premier, and MedEdPORTAL, and the searches were conducted twice. Searches were limited to English language articles. There was no restriction on year or status of publication; we included articles through November 24, 2021, in the search. Search strategies were created using medical subject headings (MeSH) and keywords combined with database-specific advanced search techniques. MeSH terms and keywords were identified to represent trauma-informed approach training for emergency and trauma care clinicians. The full search strategy from Ovid Medline is further detailed in Table 1. We downloaded a total of 6,786 results from the literature searches into EndNote, and duplicate articles were removed; 2,280 unique publications were uploaded into Rayyan (Rayyan Systems Inc, Boston, MA; https://www.rayyan.ai/) for screening (Figure 2).

**Table 1.** Ovid MEDLINE search strategy (through November 24, 2021).

1 (trauma informed or (aces or adverse child* event* or adverse child* experience*)).mp.
2 trauma.ti. or trauma.ab. or traumatiz*.mp. or traumatis*.mp. or retraumatis*.mp. or retraumatiz*.mp.
3 exp stress, psychological/ or psychological stress*.mp. or stressful event*.mp. or stressful experience*.mp. or exp life change events/ or life chang* event*.mp.
4 exp RESILIENCE, PSYCHOLOGICAL/ or resilien*.mp. or coping.mp. or cope.mp. or coped.mp.
5 exp Adaptation, Psychological/ or (psychological* adj5 adapt*).mp. or (emotional* adj5 adjust*).mp. or exp emotional adjustment/
6 exp Stress Disorders, Post-Traumatic/ or post traumatic stress disorder*.mp. or posttraumatic stress disorder*.mp. or ptsd.mp. or posttraumatic neuros*.mp. or post traumatic neuros*.mp. or (moral* adj5 injur*).mp.
7 exp social support/ or social support*.mp. or social network*.mp.
8 exp self care/ or self care.mp.
9 well being.mp. or exp “Quality of Life”/ or qol.mp. or quality of life.mp. or life quality.mp.
10 2 or 3 or 4 or 5 or 6 or 7 or 8 or 9
11 patient centered*.mp. or exp Patient-Centered Care/ or patient focused*.mp. or medical home*.mp. or client centered*.mp.
12 exp “Delivery of Health Care, Integrated”/ or (behavioral adj5 health adj5 integrat*).mp. or (behavioural adj5 health adj5 integrat*).mp. or (integrated adj5 care).mp.
13 11 or 12
14 10 and 13
15 1 or 14
16 exp education/ or exp curriculum/ or exp education, professional/ or exp education, medical/ or curricul*.mp. or ed.fs.
17 (educat* or train* or orientat* or lectur* or teach* or workshop* or pre-post or implement* or assessment*).mp.
18 exp simulation/ or simulat*.mp. or screen*.mp.
19 exp TEACHING/ or exp TEACHING MATERIALS/ or exp lectures/
20 exp Education, Medical, Continuing/ or continuing medical educat*.mp. or cme.mp.
21 exp Health Personnel/ed or interprofessional educat*.mp.
22 exp program development/ or (program* adj5 develop*).mp.
23 exp quality improvement/ or (quality adj5 improv*).mp.
24 exp Evaluation Studies as Topic/ or (research adj5 evaluat*).mp. or (program* adj5 evaluat*).mp.
25 16 or 17 or 18 or 19 or 20 or 21 or 22 or 23 or 24
26 Advanced Trauma Life Support Care/ or exp emergency medicine/ or emergency nursing/ or exp Emergency Service, Hospital/
27 (emergency or emergicenter* or emergency center* or trauma service* or trauma unit* or trauma center* or advance* trauma*).mp.
28 (emergency department* or emergency hospital service* or emergency outpatient unit* or emergency room* or emergency unit* or emergency ward* or hospital emergency service* or emergency service* or emergency nurs* or emergency physician* or emergency medicine).mp.
29 26 or 27 or 28
30 15 and 25 and 29
31 limit 30 to English language ***************************

### Study Selection

All the results were screened by three independent reviewers to determine eligibility for this review. The first phase of screening was a blinded title/abstract review conducted in Rayyan, and potentially relevant articles were moved to the second phase of screening for the full text of the publications. Conflicts were resolved with group discussion and consensus. Final analysis included identification of specific training protocols from each of the articles.

### Evaluation Criteria

Although many papers reference TIC training, we specifically sought training protocols that were described to the level that they could be duplicated and that had been evaluated with a minimal degree of rigor. Studies were selected if they met the following criteria: the population included emergency or trauma service clinicians (medical doctors, nurses, residents, nurse practitioners and physician assistants); study design involved TIC training for emergency or trauma clinicians and included evaluation of the training, and the setting was a US ED or trauma hospital environment. Only articles written in English were included.

We evaluated training protocols based on who participated, mode and length of training, evaluation methods, results, and Kirkpatrick levels (Table 2). The Kirkpatrick Model, developed in 1959, remains the most common method for evaluating the impact of training programs and is primarily used to assess medical training. As a well-established tool for evaluation, the Kirkpatrick Model is widely considered to be a valid and reliable tool that can be implemented with ease to measure the effectiveness of training on a particular target goal. The model uses four levels of training evaluation: Level 1: Reaction—how favorable, engaging, and relevant training is to the participants’ jobs; Level 2: Learning—did participants acquire the intended knowledge, skills, attitude, confidence, and commitment through participation; Level 3: Behavior—will participants apply what they learned in practice; and Level 4: Results—are targeted outcomes (changes in clinician behaviors and improved patient outcomes) due to training.^27^

**Table 2.** Summary of two approaches to trauma-informed care training for emergency and trauma physicians.

Source	Population	Training methodology	Evaluation	Results	Kirkpatrick Model level of learning
Cole R (2014)	Two new nurses and four recent graduate nurses (pilot group) at an acute-care, Level II trauma center. Expanded to include all ED staff	Four-hour pilot training course: read 2 BETA Project articles and complete homework before workshop; didactic sessions and roleplay	• Post-test • Class evaluation by pilot group	1. Most participants found the training valuable and integrable into their existing practice. 2. Initially, 15–20 episodes of restraint or seclusion per month decreased to 0 episodes. 3. Overall behavioral health seclusion/restraint hours reduced from 38.5 h/mo. (August 2011) to 0 h/mo. (September 2013) with overall shorter episode duration and improved documentation compliance.	• Reaction – Level 1 • Behavior – Level 3 • Results – Level 4
McNamara M, et al (2020)	318 clinicians and hospital staff members at two Level I pediatric trauma centers	90-minute workshops from 2015–2018 plus patient expert advice and a panel discussion, followed by implementation of “Five Points of TIC” curriculum	• Pre-/post-workshop surveys • Tracked referrals to the Violence Intervention Program (VIP) from 2014–2018	1. Increased referrals to VIP from physicians (*P* < 0.001; 7.3% to 47.8%). 2. Decreased probability of patients being identified only by VIP staff (*P* < 0.001; 62.1% to 23.4%). 3. Self-reported comfort with TIC after workshops improved by 21% (*P* < 0.001).	• Reaction – Level 1 • Behavior – Level 3

*ED*, emergency department; *BETA*, Best Practices in Evaluation and Treatment of Agitation; *h/mo*, hours/months; *TIC*, trauma-informed care.

## RESULTS

After reviewing 2,280 unique articles, we included 16 articles for full-text review. Of the 16 articles, only two were included in the final analysis. Fourteen articles were excluded for targeting the incorrect population,^23^^,^^28^ having a training center location outside the US,^29^^,^^30^ not describing the TIC training curriculum,^31^^–^^39^ or lacking evaluation of the curriculum.^40^ The included articles highlight different training protocols, one addressing the treatment of agitation and one encouraging clinician referrals.^6^^,^^7^ Both articles cover subject matter related to patient-centered communication, use in-person learning methodologies, including didactic sessions or roleplays, and address interprofessional collaboration as part of the training.^6^^,^^7^ These two articles detail the development of TIC curricula for emergency and trauma clinicians from their design to their impact, providing comprehensive insight that will be able to inform the development of future training protocols.

### Five Points of Trauma-Informed Care

One of the TIC training protocols, entitled the Five Points of TIC, was implemented for Level I trauma center clinicians.^7^ Clinicians and staff from the departments of EM, pediatrics, surgery, and social work, as well as medical students and nurses, among others, participated in training that consisted of a 90-minute workshop, facilitated by a pediatrician and former patients. This model outlined five pillars to guide clinicians and aid families affected by trauma or violent injury: safety; screening; understanding context; avoiding re-traumatization; and discharge planning. Additionally, the training focused on promoting a patient’s sense of safety, which can help improve their healing and establish trust between clinician and patient.^7^ This includes factors such as privacy, a consistent and dependable clinician, and a soothing environment.

Within this workshop, participants discussed correlating clinical cases, complex trauma, and a hospital-based violence intervention program (VIP). The VIP included trauma intervention specialists who could provide crisis intervention, support, and psychoeducation on trauma. Next, participants reviewed the Five Points of TIC and discussed patient cases. Following the cases, they held a patient panel and VIP panel, where patients were able to share their experiences with trauma-sensitive communication skills and healing.

The Kirkpatrick levels highlighted for this protocol include Levels 1 and 3. Participants completed pre- and post-workshop surveys assessing comfort with the Five Points of TIC. Results demonstrated an increase in comfort levels with TIC (*P* < .001) for attendings, residents, fellows, and medical students, with medical students having the highest increase in comfort levels (Level 1). Additionally, behavioral change was directly assessed, with VIP referrals from physicians significantly increasing from 7.3% in 2014 to 47.8% of patients referred in 2018 following the course (*P* < .001) (Level 3). These results demonstrate that as a result of training, there can be an improvement in TIC comfort and familiarity with TIC approaches, leading to substantive change in practice.

### BETA Project

Another TIC training protocol was completed by nurses, and later all staff, in the ED.^6^ Participants completed Management of the Agitated Patient in the Emergency Department training, part of the Best Practices in Evaluation and Treatment of Agitation (BETA) project, which focuses on evidence-based guidelines and non-pharmacological interventions to minimize use of restraints and seclusion when caring for agitated patients. De-escalation techniques, environmental modifications, and sensory approaches are the foundation of this approach.

The four-hour training consisted of didactic simulations and role play. Beyond staff education, the protocol also called for the development of new clinical processes and ongoing monitoring and feedback. Based on the Kirkpatrick Model of training evaluation, learning associated with this protocol included Levels 1, 3, and 4. Following completion of the training, results indicated that the nurses found it valuable and able to be easily integrated into their practice (Level 1). Participants reported improved confidence and satisfaction with managing aggressive patients (Level 1). There was also a significant reduction in restraint use in the ED, demonstrating that a behavioral change and improved outcomes can occur through providing staff with TIC knowledge and the skills to address underlying causes of patient behaviors (Level 3 and 4).

## DISCUSSION

This review highlights the need for continued development and evaluation of outcomes of TIC trainings for emergency and trauma service physicians. Although only two curricula were identified that met the inclusion and exclusion criteria established for this review, several studies highlighted the importance of TIC training (Hawkins, Fisher).^9^^,^^34^ These studies do not, however, include specific curricula that were used to train emergency and trauma service physicians. To promote the literature on this topic and aid institutions striving to bring TIC to their EM or trauma services, it is important to not only identify the training curricula available for emergency and trauma service clinicians, but to evaluate the *effectiveness* of the TIC training.

Prior to designing and implementing a training protocol, a needs assessment can be conducted to determine the specific deficits within an institution or practice.^21^^,^^41^ This is a step that was not indicated in the current included results and may be an important piece prior to creating a curriculum.^6^^,^^7^ To create the most impactful curriculum, the needs of the clinicians, patients, and communities must be understood. First, this involves surveying clinician attitudes and beliefs about TIC, as well as specific knowledge of what TIC encompasses and its role in building trust within the medical system.^8^ Second, this involves asking clinicians what they feel they may need in TIC training and the outcomes they are hoping for.

The needs as perceived by physicians on a trauma service may differ dramatically from those as perceived by outpatient primary care physicians.^21^ Furthermore, a needs assessment would promote understanding of any TIC approaches that are already being implemented (whether or not they are explicitly recognized as TIC) within the ED or trauma service setting. Finally, this needs assessment would focus on addressing concerns of the unique patient populations that the clinicians care for. Even across EDs and trauma services, there may be marked differences in patient populations and community resources already available, which may impact what is emphasized in a hospital-based TIC training.

When developing a training protocol, outcomes have indicated that even moderate training improves the ability of the healthcare team to provide TIC; however, more intensive protocols are correlated with improved results.^35^ Protocols such as the Five Pillars of TIC and the BETA Project, which use in-person workshops with case-based discussions, role-play, and simulations versus didactics alone, show greater clinician proficiency associated with improved patient-reported outcomes and physician comfort levels.^6^^,^^7^^,^^35^

Although the literature regarding the outcomes of implementing TIC training for emergency and trauma service physicians is limited, research on the development of such training programs suggests that training and simulations should encourage a multidisciplinary approach, mirroring the reality of the environment.^41^ This method helps both to identify system-level conditions that might impact the delivery of TIC, such as organizational issues, and to highlight any social dynamics or authority hierarchies that could discourage team members from voicing concerns.

As noted in the included articles by Cole (2014) and McNamara et al (2020), the success of a TIC protocol can be evaluated through pre- and post-training surveys or evaluations to gauge the impact of the course on healthcare clinicians and their practice.^6^^,^^7^^,^^35^ Metrics that include referral to outside resources, involvement of social workers, and patient satisfaction can be used to track successful implementation of TIC methods as illustrated through the BETA Project.^7^ These can be monitored by monthly or quarterly chart audits and patient surveys.^6^ Additionally, long-term evaluation of behavioral changes, knowledge and beliefs, and comfort with providing TIC should be tracked to monitor the impact of the training program.

Finally, future research should emphasize the ways in which TIC can improve healthcare costs, clinician satisfaction and well-being, and long-term health outcomes for patients affected by traumatic experiences, including reduced re-traumatization, decreased healthcare utilization, improved mental and physical health outcomes, and decreased substance use.^19^ Existing evidence suggests that recognizing trauma’s impact on patient behavior and health allows clinicians to avoid unnecessary interventions, decrease readmissions, and improve health outcomes.^5^ Additionally, evaluating TIC practices to reduce clinician burnout could limit staff turnover and associated recruitment and training costs.^10^^,^^23^^,^^24^ This data, along with more robust data from emergency and trauma services that have implemented TIC protocols, is critical in ultimately providing the most considerate and appropriate care for patients.

## LIMITATIONS

This review is limited in that only two articles were found to meet inclusion criteria. While there was more available research on TIC training evaluations within mental health and primary care settings, the unique nature of EDs and trauma services warranted strict inclusion criteria, which resulted in a narrow selection of literature. In these settings, patients are faced with unfamiliar physicians and fast-paced interactions, and there is evidence indicating that a large proportion of patients in these settings report a history of trauma and often rely on acute care for all healthcare needs.^11^^,^^12^^,^^15^^,^^16^ An environment emphasizing empathy and safety is paramount in TIC, especially in these departments.^7^^,^^10^ The primary intention of including evaluations of TIC training only in US healthcare facilities was to account for differences in healthcare system resources and investment in training compared to other countries. Excluded were articles discussing the potential of certain TIC training and practices without evaluation of effectiveness that would inform future curricula development. With the necessary criteria that were established for a robust review, the final results yielded limited data for determining the most optimal features of a TIC training protocol.

## CONCLUSION

Our review demonstrates a considerable paucity in the literature regarding implemented and evaluated trauma-informed care curricula for emergency and trauma service clinicians. However, the existing training protocols demonstrate that, with targeted training, clinicians become more comfortable with TIC and can integrate aspects of TIC into current practices.
